# Should Nurses Live out?

**Published:** 1918-09-21

**Authors:** 


*To the Editor of* THE HOSPITAL.

### Should Nurses Live Out?

*To the Editor of* THE HOSPITAL.

SIR,—I have read with interest, but not with pleasure, "Ierne's" letter of August 31, and feel sure that if she be a woman she has no practical experience of the nursing world. I think it more probable that "Ierne" is a man and consequently knows nothing of the profession other than what he has heard from "partially-trained" nurses, or nurses of the standard he refers to—"a lower class of woman, on a level with the domestic servant." If the class has necessarily had to be lowered, is it not our duty and our aim to raise the standard of nursing? Nurses are not miners or factory hands, and have no wish for strikes or trade unions, nor do they, like the shop-girl, wish to "live out." I should be sorry for the hospitals and other institutions if the nurses "lived out." On the least pretext, such as slight headache, or in cold, wet, or snowy weather, she would not "turn up" in the morning, and in this case the patients would suffer. I do not know of any nurse who would care to turn out at night or in the early morning. They are made very comfortable in most institutions, are given every attention if they complain of not feeling well, and have plenty of freedom when "off duty." If nurses have a grievance—which I don't think they have, at any rate not that which "Ierne" refers to—they will leave it until after the war, as all are anxious to help win the war, and do not wish to delay our final victory by wasting time and paper.

No careful mother would allow her daughter to leave home to enter a hospital or infirmary where she had to "live out." A dearth of the "right" kind of nurse would be inevitable.

A FULLY-TRAINED "LONDON" HOSPITAL NURSE.

(At present night superintendent in a large infirmary.)

September 9.

